# *IRS1* gene variants, dysglycaemic metabolic changes and type-2 diabetes risk

**DOI:** 10.1016/j.numecd.2011.05.009

**Published:** 2012-12

**Authors:** N. Yiannakouris, J.A. Cooper, S. Shah, F. Drenos, H.A. Ireland, J.W. Stephens, K.-W. Li, R. Elkeles, I.F. Godsland, M. Kivimaki, A.D. Hingorani, M. Kumari, P.J. Talmud, S.E. Humphries

**Affiliations:** a Harokopio University of Athens, 17671 Athens, Greece; b Centre for Cardiovascular Genetics, Department of Medicine, Rayne Building, Royal Free and University College Medical School, 5 University Street, London WC1E 6JF, UK; c University College London Genetics Institute, Department of Genetics, Environment and Evolution, Gower St, London WC1E 6BT, UK; d Diabetes Research Group, Institute of Life Sciences, School of Medicine, Swansea University, Swansea SA2 8PP, UK; e Endocrinology and Metabolic Medicine, Imperial College London and St Mary’s Hospital, London W2 1NY, UK; f Genetic Epidemiology Group, Department of Epidemiology and Public Health, University College London, 1-19 Torrington Street, London WC1E 6BT, UK

**Keywords:** *IRS1*, GWAS, Genetic variation, Type-2 diabetes, Hyperinsulinemia, Insulin resistance, IRS1, Insulin receptor substrate-1, T2D, Type-2 diabetes, CVD, Cardiovascular disease, GWAS, Genome-wide association studies, SNP, Single nucleotide polymorphism, HOMA-IR, Homeostasis model assessment of insulin resistance, OGTT, Oral glucose tolerance test, LD, Linkage disequilibrium, WHII, Whitehall-II, NPHSII, Northwick Park Heart Study-II, UDACS, UniversityCollege London Diabetes and Cardiovascular Study, EDS, Ealing Diabetes Study, PREDICT, PRospective Evaluation of Diabetic Ischemic heart disease by Computed Tomography, EARSII, European Atherosclerosis Research Study-II, DIAGRAM, Diabetes Genetics Replication and Meta-analysis Consortium

## Abstract

**Background and aims:**

A recent genome-wide association study identified rs2943641C > T, 500 kb from the insulin receptor substrate-1 gene (*IRS1*), as a type-2 diabetes (T2D) susceptibility locus. We aimed to replicate this association by meta-analysis and examine whether common variants within *IRS1*, present on the HumanCVD BeadChip, were associated with T2D risk.

**Methods and results:**

We genotyped rs2943641 in 2389 prevalent or incident T2D patients and 6494 controls from two prospective and three case studies based in UK and in the European Atherosclerosis Research Study-II (EARSII; *n* = 714). Thirty-three *IRS1* variants had been genotyped in the prospective Whitehall-II study (*n* = 4752) using the HumanCVD BeadChip. In a fixed-effects meta-analysis of the UK study cohorts rs2943641T allele was associated with 6% lower risk of T2D (*p* = 0.18), with T-allele carriers having an odds ratio (OR) of 0.89 (95% confidence interval [CI]: 0.80–1.00, *p* = 0.056) compared to CC subjects. The T-allele was also associated with lower fasting insulin and homeostasis model assessment index of insulin resistance in Whitehall-II and with lower post-load insulin after an oral glucose tolerance test in EARSII (all *p <* 0.05). None of the *IRS1* variants on the chip showed linkage disequilibrium with rs2943641. *In silico* analysis with follow-up genotyping (total *n* = 9313) identified that the rare allele of the *IRS1* promoter variant rs6725556A > G showed association with reduced T2D risk (OR per G-allele: 0.82, 95%CI: 0.69–0.96, *p* = 0.015).

**Conclusions:**

We confirm the association of rs2943641T with T2D protection. There is a possible independent effect on risk of a putative *IRS1* promoter variant.

## Introduction

Genome-wide association studies (GWAS) have identified multiple loci at which common variants modestly but reproducibly influence risk of type-2 diabetes (T2D) [1–6]. Currently, single nucleotide polymorphisms (SNPs) in ∼40 genetic loci have been associated with T2D [7,8], most of which relate to insulin secretion rather than insulin resistance [8,9], have been distinct from previously studied candidate genes [10], and do not seem to offer greater predictive value in determining diabetes risk than do commonly used phenotypic risk factors and family history [11,12].

Rung and colleagues [13] identified rs2943641C > T, located 500 kb downstream of the insulin receptor substrate-1 gene (*IRS1*), as a T2D risk locus, with the major C-allele being associated with 19% increased risk of T2D. Importantly, unlike other reported T2D loci, the rs2943641C allele was associated with increased fasting- and glucose-stimulated hyperinsulinemia and impaired insulin sensitivity. Lower IRS1-associated phosphatidylinositol-3–OH kinase activity in human skeletal muscle biopsies was also shown for the C-allele during insulin infusion, and *in vitro* studies showed that this allele was associated with lower IRS1 protein expression in the basal state, suggesting a direct regulatory link between rs2943641 and IRS1 [13]. The Diabetes Genetics Replication and Meta-analysis Consortium (DIAGRAM) in an earlier meta-analysis did not identify this SNP as a T2D risk variant [4]; however, in a subsequent publication [6] a different *IRS1* SNP (rs7578326) adjacent to and in strong linkage disequilibrium (LD) with rs2943641 (*r*^2^ = 0.79, in HapMap CEU) was reported to be associated with T2D.

The purpose of this study was to validate the rs2943641 association with T2D risk and diabetes-related quantitative traits using data from UK population-based cohorts and T2D patients. In addition, using data from 4752 Caucasians participating in the Whitehall-II study who had been genotyped for 33 *IRS1* SNPs using the HumanCVD BeadChip [14,15] and with follow-up direct genotyping of *IRS1* SNPs in the other study cohorts, we explored the potential association with the risk of T2D of SNPs within and flanking *IRS1*.

## Methods

### Study cohorts

Full details of the 6 studies, Whitehall-II (WHII), Northwick Park Heart Study-II (NPHSII), University College London Diabetes and Cardiovascular Study (UDACS), Ealing Diabetes Study (EDS), PRospective Evaluation of Diabetic Ischemic heart disease by Computed Tomography (PREDICT) and European Atherosclerosis Research Study-II (EARSII) are presented in the Supplementary Methods section and in Supplemental Table 1. All studies were approved by their institutional ethics committees and all subjects gave written informed consent.

### Insulin resistance and HbA1c determination

In EARSII and WHII insulin resistance estimates were derived using the homeostasis model assessment index of insulin resistance (HOMA-IR) = fasting insulin (pmol/l) × fasting glucose (mmol/l)/156.3 [16]. Insulin sensitivity and β-cell function were also accessed using the oral glucose tolerance test (OGTT) [17]. HbA1c was measured in EDTA-whole blood on a calibrated high-performance liquid chromatography system with automated haemolysis before injection [18].

### Genotyping

*In silico* data for SNPs spanning *IRS1* was obtained from WHII where genotyping had been undertaken using the 50K-HumanCVD BeadChip (Illumina, San Diego, USA) [14,15]. Thirty-three SNPs present on the chip, located either in coding, non-coding, or in the flanking region of *IRS1* (within 5 kb upstream or downstream of the gene), were considered. Ten SNPs were monomorphic in WHII, while for the rest, minor allele frequencies among T2D-free individuals were in the range of 0.023–0.111 (Supplementary Table 2). Direct genotyping of rs2943641 in all cohorts and of *IRS1* rs6725556 in other study cohorts was carried out using TaqMan on the ABI-7900HT platform (Applied Biosciences, Warrington, UK). Random duplicates were used as quality control with call rates >96%. In all studies, genotype distribution was as expected from Hardy-Weinberg proportions.

### Statistical analysis

For continuous variables results are presented as mean ± SD. Non-normally distributed variables were logarithmic or square-root transformed and means were transformed back and SDs are approximate for these variables. In WHII, glucose, insulin, HOMA-IR, HbA1c, systolic-blood pressure (BP), diastolic-BP and body mass index (BMI) were log-transformed. In EARSII, insulin values were square-root transformed and cholesterol, BMI, and systolic-BP were log-transformed. *P*-values are adjusted for covariates using analysis of covariance models. Categorical variables are presented as percentage and number, and are compared using chi-squared tests. Glucose, insulin and HOMA-IR were compared using data from all phases in WHII (phases 3, 5 and 7) using multi-level mixed regression (random-intercept model). Adjustment was made for age, BMI and gender, and dummy variables were fitted for phase-5 and phase-7 in order to take account of differences in measurements over time. Diabetes status, as the outcome, was analysed by logistic regression with adjustment for age and where applicable for gender and recruitment centre. Results for rs2943641 and rs6725556 were combined over all cohorts using fixed-effects meta-analysis. In WHII a set of non-redundant *IRS1* SNPs independently associated with T2D was determined by variable selection, using stepwise regression based on the Bayesian information criterion [19]. An additive genetic model was assumed. Of the 23 SNPs, 18 with *p* < 0.25 on univariate analysis were initially selected for possible inclusion in the multivariate model. Statistically significance was taken as *p* < 0.01. Following the suggestion of Rothman [20], this more conservative *p*-value was used in preference to correcting for multiple comparisons.

## Results

### Association of rs2943641 with diabetes-related quantitative traits

Baseline clinical, biochemical, and the genetic characteristics of the subjects in WHII and NPHSII are presented in Supplementary Table 3. Subjects who went on to develop T2D were more likely to be obese and hypertensive, and in WHII had, as expected, higher baseline fasting glucose and insulin levels, higher percentage of HbA1c and a higher HOMA-IR index (all *p* < 0.001). There were no significant genotype differences between T2D cases and controls; however, in WHII the rs2943641T allele was associated with lower fasting insulin (*p* *=* 0.04) and HOMA-IR (*p* = 0.03) in a mixed regression model over all study phases while adjusting for age, gender, BMI and study phase (Supplementary Table 4).

The overall characteristics of the T2D patients in UDACS, EDS and PREDICT by ethnic group and rs2943641 genotype, are presented in Supplementary Tables 5 and 6. In comparison to European whites, patients of Indian Asian origin had an earlier age of onset of the disease, a lower prevalence of obesity and were less frequently smokers and carriers of the rs2943641T allele (Supplementary Table 5). No differences in any baseline biochemical measures, including fasting glucose and HbA1c, were observed across genotypes in the two ethnic groups (Supplementary Table 6).

**Figure 1:**
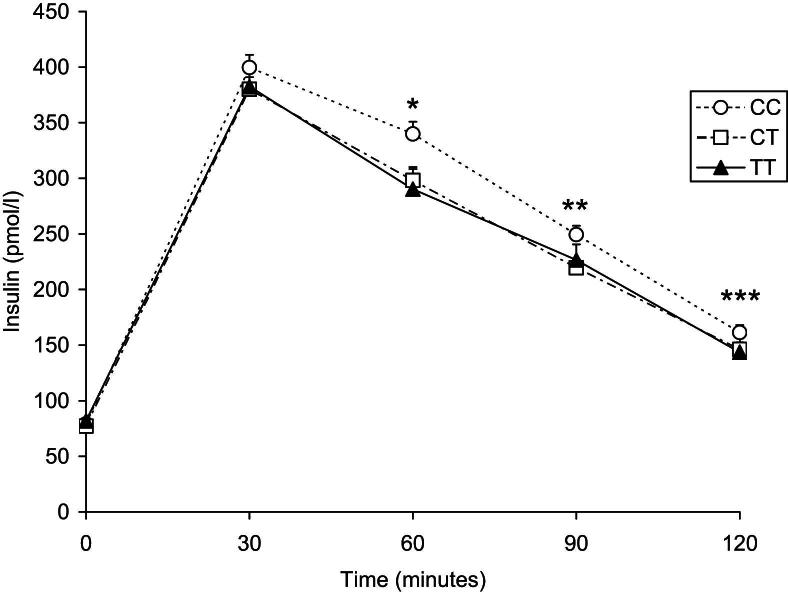
Plasma insulin concentration after an OGTT by rs2943641 genotype in European Atherosclerosis Research Study-II. Data are presented as mean ± SEM in carriers of the CC (white circles), CT (white squares) and TT (black triangles) genotypes. ∗*p* = 0.004, ∗∗*p* = 0.03, ∗∗∗*p* = 0.09, for additive model adjusting for age, “case”/“control” status and region. *P*-values for AUC insulin: *p* = 0.005 (additive), *p* = 0.003 (dominant), confirming result reported by Rung et al. [13].

In EARSII, there was no ‘case’/‘control’ heterogeneity in age, BMI, BP, fasting glucose or rs2943641 genotype distribution (Supplementary Table 3) and therefore, ‘cases’ and ‘controls’ were combined in subsequent analyses. No significant differences across genotypes for any of the fasting biochemical variables were observed in this cohort of young individuals; however, rs2943641T allele was associated with lower insulin levels after OGTT (Fig. 1). The effect of rs2943641T appeared to be dominant, with T-allele carriers having area under the curve (AUC) for insulin 13.3% lower than CC homozygotes (*p* = 0.003). The difference among genotypes was significant at 60 and at 90 min after the OGTT (*p* = 0.004 and *p* = 0.03, respectively, Fig. 1). There was no evidence for heterogeneity between ‘cases’ and ‘controls’ for AUC_insulin_ (*p* = 0.47), nor were any differences between genotype groups for AUC_glucose_ (Supplementary Table 7). OGTT-derived indexes of insulin sensitivity as reflected by metabolic clearance rate (MCR) and the insulin sensitivity index (ISI), and β-cell function as reflected by 1st and 2nd phase insulin release [17], showed that both MCR and ISI were significantly associated with rs2943641 genotype (Table 1).

### Impact of rs2943641 on T2D risk

**Figure 2:**
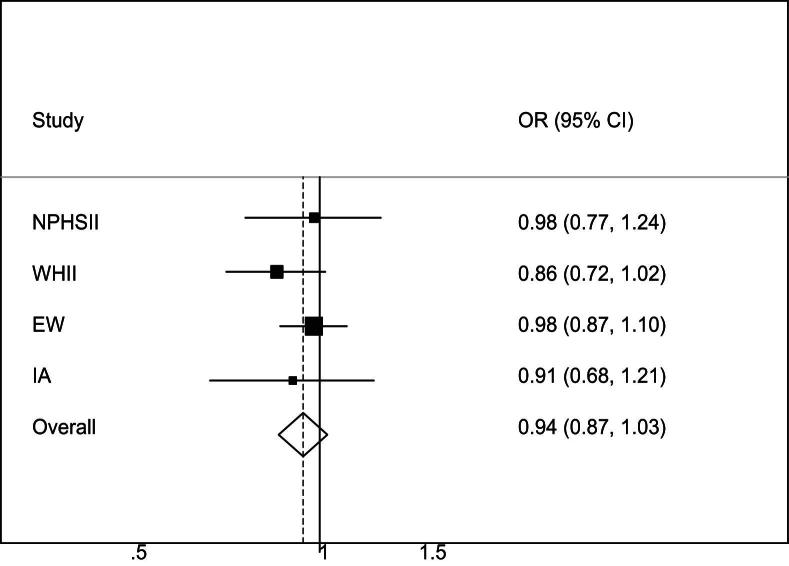
Odds ratio (OR) and 95% confidence interval (CI) for T2D per rs2943641T allele (additive genetic model) in the various populations of this study. Heterogeneity chi-squared = 0.73 (d.f. = 3), *p* = 0.87; I-squared (variation in OR attributable to heterogeneity) = 0.0%. ORs were adjusted for age and where applicable for gender and centre. *Abbreviations:* NPHSII = Northwick Park Heart Study-II; WHII = Whitehall-II; EW = European Whites (UDACS, EDSC and PREDICT combined); IA = Indian Asians (UDACS, EDSC and PREDICT combined).

The association of rs2943641 with T2D in the study populations was examined using logistic regression with adjustment for age plus gender and general practice recruitment where applicable. For European white T2D patients, the NPHSII T2D-free group was used for comparison (*n* = 2489), whereas for Indian Asians the WHII Asian non-diabetic participants (*n* = 146) were used. Fig. 2 shows odds ratios (ORs) and 95% confidence intervals (CI) for T2D for each additional T-allele carried (additive effect). Overall, the rs2943641T allele was associated with a 6% decreased risk of T2D (*p* = 0.18 for an additive model), with T-allele carriers having a OR of 0.89 (95%CI: 0.80–1.00, *p* = 0.056) compared to CC subjects.

### Association of *IRS1* SNPs with T2D risk in WHII

The association of the *IRS1* SNPs on the HumanCVD BeadChip (used to genotype in WHII) with T2D risk are presented in Supplementary Tables 2 and 4. None of these SNPs were in LD with rs2943641 using data from HapMap PhaseIII for the CEU population, or in WHII, although strong LD (*r*^2^ > 0.6) between several *IRS1* SNPs was observed in WHII (Supplementary Fig. 1). Eight *IRS1* SNPs showed suggestive evidence for an association of the minor alleles with a decreased risk of T2D (*p*-values *≤*0.05), but no association with diabetes-related quantitative traits, including fasting and 2-h after OGTT glucose and insulin concentrations, were observed (Supplementary Table 4). Seven of these SNPs represent two LD-blocks within *IRS1* (Supplementary Figure 1), while rs6725556 is located 3538 nucleotides upstream of the *IRS1* translation start site.

Using a variable selection model including all risk-associated *IRS1* variants, age, gender and BMI considered for entry, we identified that rs6725556 (OR per minor G-allele: 0.50, 95%CI: 0.33–0.78, *p* = 0.002) and SNP rs2943641 near *IRS1* (OR per minor T-allele: 0.82, 95%CI: 0.69–0.99, *p* = 0.04) were the only variants that were independently associated with T2D risk in WHII, along with age (*p* < 0.001) and BMI (*p* < 0.001). The two SNPs appeared to have an additive effect (logistic scale) on risk with no statistically significant evidence for interaction (*p*_interaction_ = 0.15). Considering subjects homozygous for rs2943641C as the reference category, the risk of being an incident T2D case was lower in carriers of both minor alleles of the rs2943641 and rs6725556 polymorphisms (OR: 0.48, 95%CI: 0.27–0.84, *p* = 0.01) compared to carriers of the rs2943641T allele alone (OR: 0.70, 95%CI: 0.54–0.90, *p* = 0.006).

### Impact of *IRS1* rs6725556 on T2D risk

**Figure 3:**
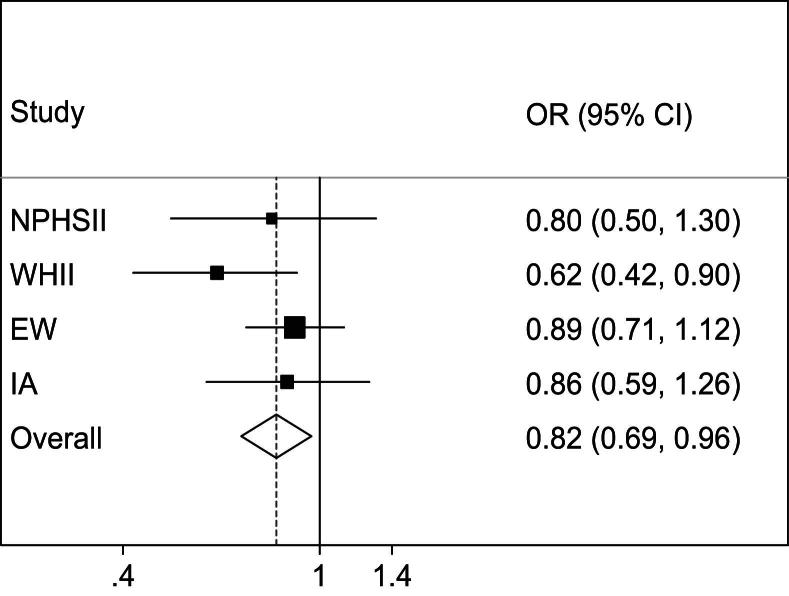
Odds ratio (OR) and 95% confidence interval (CI) for T2D per *IRS1* rs6725556G allele (additive genetic model) in the various populations of this study. Heterogeneity chi-squared = 4.36 (d.f. = 3), *p* = 0.23; I-squared = 31.1%. ORs were adjusted for age and where applicable for gender and centre. *Abbreviations*: NPHSII = Northwick Park Heart Study-II; WHII = Whitehall-II study; EW = European Whites; IA = Indian Asians.

To assess further the impact of rs6725556A > G on T2D risk we genotyped this SNP in NPHSII and in the T2D patients (Supplementary Table 8). Compared to European white T2D patients, the frequency of the variant rs6725556G allele was twice as high in T2D patients of Indian Asian origin (0.06 vs. 0.12, respectively, *p* < 0.001). In all UK study cohorts, there was a trend for an association of the G-allele with decreased risk for T2D. Meta-analysis of individual participant data from all UK cohorts (Fig. 3) revealed that the rs6725556G allele was associated with lower risk of T2D (OR per G-allele: 0.82, 95%CI: 0.69–0.96; *p* = 0.015).

## Discussion

We genotyped rs2943641C > T, located 500 kb downstream of *IRS1*, in 2389 prevalent or incident T2D patients and 6494 controls from two prospective and three case studies based in UK and found evidence for an association of the minor rs2943641T allele with T2D protection. This allele was associated with lower fasting insulin and HOMA-IR index in middle-aged participants of the WHII study and with lower post-load insulin after OGTT in young adults of the EARSII study. *In silico* analysis with follow-up genotyping also identified that the minor allele of the *IRS1* promoter variant rs6725556A > G showed association with reduced T2D risk (OR per G-allele: 0.82, 95%CI: 0.69–0.96, *p* = 0.015).

Rung and colleagues [13] identified rs2943641 as a T2D susceptibility locus in a multistage association study across 14,051 French and Danish individuals (6258 cases and 7793 controls) and showed strong association of the major C-allele with increased risk of T2D (OR: 1.19, 95%CI: 1.13–1.25, *p* = 9.3 × 10^−12^). This result is equivalent to OR per T-allele: 0.84, 95%CI: 0.80–0.88. Our findings in these UK studies are consistent with an association of rs2943641T with 6% decreased risk of T2D (OR per T-allele: 0.94, 95%CI: 0.87–1.03, *p* = 0.18). This association became statistically significant when analyses were repeated with additional adjustment for BMI (overall OR: 0.88; 95%CI: 0.80–0.96, *p* = 0.006), although since there was no relationship of this SNP with BMI, and GWAS of genetic variants influencing BMI, obesity and related phenotypes have not identified *IRS1* as a BMI related gene [8], the mechanism of this is unclear.

Notably, data from the recently published DIAGRAM meta-analysis [6] identified a different SNP (rs7578326A > G) adjacent to rs2943641 to be associated with T2D (OR per A-allele: 1.11, 95%CI: 1.08–1.13, *p* = 5.4 × 10^−20^; 42,542 cases and 98,912 controls). The two SNPs lie ∼73 kb apart and are in strong LD (*r*^2^ = 0.79 in HapMap CEU), and therefore this finding provides further confirmation of the previously reported signal. Moreover, using data from up to 46,186 non-diabetic subjects from the Meta-Analyses of Glucose and Insulin-related traits Consortium the authors reported the risk allele to be associated with higher fasting insulin [6], consistent with a primary effect on insulin action.

Rung and colleagues [13] also examined the effect of rs2943641 on diabetes-related quantitative traits in three independent cohorts with normoglycemic individuals of Finnish, French and Danish origin (*n* = 14,358) and found that the diabetogenic rs2943641C allele was associated with higher fasting insulin and HOMA-IR indices, but not with fasting glucose levels. In middle-aged Danes, the C-allele was also associated with higher insulin levels after OGTT [13]. Concordant with those findings, we found an association of the minor rs2943641T allele with lower fasting insulin (*p* = 0.04) and HOMA-IR (*p* *=* 0.03) in middle-aged individuals of WHII study, and no association with fasting glucose levels. We have also taken this study forward by examining the association of this variant with insulin levels after an OGTT. The T-allele was associated with lower post-load insulin levels in the healthy young males of EARSII (13.3% lower AUC_insulin_, *p* = 0.003), but it was not associated with fasting insulin or HOMA-IR, which implies that a significant association of rs2943641T with lower fasting insulin levels may be evident or become established only later in life.

The importance of *IRS1* in insulin signaling has been confirmed in studies showing that this gene is associated with peripheral insulin sensitivity as well as in the regulation of insulin secretion [21,22] and a functional *IRS1* variant (Gly972Arg, rs1801278) has been related to T2D risk [23], although some studies have failed to replicate this [24,25]. Rs1801278 was present on the 50K-chip [14] and in WHII it did not show significant association with T2D risk (OR: 1.20, 95%CI: 0.88–1.63, *p* = 0.25). Morini and colleagues [26] in their meta-analysis of 32 studies suggest that when analysis took into account age of onset of disease (data from 14 studies) there was evidence that this variant was more strongly associated with risk in the tertile of those who had early-onset disease. Our results support this (Supplementary Table 9) but our study was not powered to find a significant interaction (*p* = 0.15).

In our exploration of T2D risk as a function of 23 polymorphic *IRS1* variants on the HumanCVD BeadChip using data from WHII [12,15], with follow-up genotyping in other study cohorts, we found evidence for a possible independent effect on risk of a genetic variant in the 5′-flanking region of *IRS1* (rs6725556; −3538A > G), although no test met our prespecified criteria of *p* < 0.01 for statistical significance. Specifically, the G-allele of rs6725556 was associated with 18% lower risk of T2D in a meta-analysis of individual participant data from all UK-study cohorts (*p* = 0.015). This variant, together with rs2943641 near *IRS1*, was independently associated with T2D risk in WHII using a variable selection model with adjustment for age, gender and BMI (OR: 0.50, 95%CI: 0.33–0.78, *p* = 0.002 and OR: 0.82, 95%CI: 0.69–0.99, *p* = 0.04, respectively) and appeared to have an additive effect on risk. No corrections have been made for multiple comparisons and so these effects should be interpreted cautiously. Rs6725556 and rs2943641 lie 573.5 kb apart, and show no LD (*r*^2^ = 0.0 in WHII) and although they were both associated with risk of T2D, rs6725556 was not associated with fasting and post-load insulin levels and HOMA-IR. Interestingly, transcription factor binding site analysis using the MatInspector software tool (http://www.genomatix.de/products/MatInspector/) revealed that rs6725556G abolishes a PAX-2/5/8 binding site. The paired-box (PAX) gene family encodes a group of transcription factors that have emerged as important regulators of organogenesis in all species [27] and PAX2 has been shown to be expressed in endocrine pancreas where one of its functions may be the regulation of pancreatic hormone genes [28]. This could be of relevance in the pathogenesis of diabetes and other endocrine disorders; however, whether rs6725556 is indeed a functional polymorphism affecting *IRS1* expression needs to be proven in future functional studies. Moreover, we acknowledge that these results are preliminary and that replication of our findings in independent cohorts is essential. We also acknowledge that a limitation of our study is that it is underpowered to detect an association in the Indian Asian cohort. We only have 24% power to detect the association found by Rung and colleagues [13] (OR = 0.84) for rs2943641. However, for the Whites we have 99% power to detect a OR of 0.84. If we take account of multiple comparisons for the 6 traits (Supplementary Table 4) we would still have 94% power.

In summary, this report confirms the association of the major C-allele of rs2943641 near *IRS1* with increased risk of T2D, fasting- and glucose-stimulated hyperinsulinemia and impaired insulin sensitivity. Our data also suggest that rs2943641 and an *IRS1* putative promoter variant (rs6725556) may independently influence T2D risk, although further studies with larger cohorts are needed to confirm the etiological SNPs and to analyze their interactions in different populations.

## Figures and Tables

**Table 1:** OGTT-derived indexes of insulin sensitivity, metabolic clearance rates, and first and second phase insulin release based on Strumvoll et al [17]in EARSII.

rs2943641 genotype	Insulin sensitivity index	*p-*value^a^	Metabolic clearance rate	*p-*value^a^	First phase insulin release	*p-*value^a^	Second phase insulin release	*p-*value^a^
CC	0.118 (0.001)		9.96 (0.072)		1225.2 (24.3)		324.3 (5.3)	
CT	0.121 (0.001)	0.008	10.21 (0.071)	0.008	1197.8 (23.4)	0.83	317.7 (10.4)	0.72
TT	0.122 (0.002)		10.29 (0.139)		1235.7 (47.3)		325.0 (10.4)	

Abbreviations: OGTT = oral glucose tolerance test; EARSII = European Atherosclerosis Research Study-II.

Results are mean ± SEM.

a *P*-values adjusted for age, ‘case’/‘control’ status and region.
