# Supplementary material for: IRS1 gene variants, dysglycaemic metabolic changes and type-2 diabetes risk
Source: Nutr Metab Cardiovasc Dis. 2012 Dec;22(12):1024–30. doi: 10.1016/j.numecd.2011.05.009 (PMC3657179; doi:10.1016/j.numecd.2011.05.009)
Supplement: Supplementary file 1 [file mmc1.doc]

**Supplementary Data**

**Supplementary Methods**

**Study cohorts**

*Whitehall-II study (WHII)*

Between 1985 and 1988, all civil servants aged between 35 and 55 years in 20 departments in central London were invited to a medical examination at their workplace [1]. With 73% participation, the cohort included 10,308 participants at entry to the study. At phase 3 in 1991-3, all participants were invited to the screening clinic, which included a 75 g oral glucose tolerance test (OGTT). Biochemical screening was repeated at phase 5 (1997-9) and phase 7 (2003-4). Of the baseline participants, 6,156 participated in phase 7 screening, and of these individuals 5,666 had DNA samples. Diabetes was defined as prevalence at phase 7 according to World Health Organization (WHO) criteria by a 2-hr glucose of at least 200 mg/dl, fasting glucose of at least 126 mg/dl, use of diabetes medication or self-report of doctor diagnosis [2]. For association studies with traits only European white Caucasian subjects were used (n=4,752), while for case-control genotype and allele frequency comparisons 148 subjects of self-reported South Indian origin were used as diabetic-free controls (Supplementary Table 1).

*Second Northwick Park Heart Study (NPHSII)*

The study consists of 3,012 unrelated European white men aged 50-64 years, recruited from 9 UK general practices and followed prospectively for 15 years [3]. T2D was diagnosed based on WHO guidelines [2]. New cases were identified up to the end of 2005 by practice note search for physician diagnosed and treated T2D according to current national guidelines. Full details have been published elsewhere [3]. Of 2,705 participants with DNA samples free from diabetes at baseline, 2,682 were genotyped for at least one of the SNPs (Supplementary Table 1).

*University College London Diabetes and Cardiovascular Study (UDACS), Ealing Diabetes Study (EDS) and PRospective Evaluation of Diabetic Ischemic heart disease by Computed Tomography study (PREDICT)*

UDACS and EDS are cross-sectional samples of individuals with T2D according to WHO criteria (2), designed to study the association between common gene variants and biochemical risk factors implicated in coronary heart disease (CHD) in patients with diabetes. T2D subjects were those presenting with diabetes at 40 years or over and not requiring insulin therapy within 12 months of diagnosis. The full characteristics of the patients have been reported previously [4,5]. UDACS comprised 600 European white and 107 Indian Asians, of which 570 and 101 had genotyping for at least one of the SNPs, respectively. In EDS there were 331 European white and 503 Indian Asians, with genotyping available for 326 and 488, respectively (Supplementary Table 1).

PREDICT is a prospective study of patients with established T2D, recruited from routine diabetes clinics at London hospitals to assess the predictive value of coronary artery disease classification by electron beam tomography for CHD events and stroke [6]. Four-hundred European whites and 118 Indian Asians were considered for the present analysis, of which 395 and 114 had genotyping for at least one of the SNPs (Supplementary Table 1).

*European Atherosclerosis Research Study-II (EARSII)*

Participants of EARSII, all male aged 18-28 years, were recruited on the basis of their father having had a proven myocardial infarction before the age of 55 years and were termed ‘cases’ (n=407) and age-matched ‘controls’ (n=415), with the aim of comparing postprandial responses (for further details, see [7]). Of these individuals 779 had DNA samples and 742 were genotyped for at least one of the SNPs (Supplementary Table 1). A standard 75 g OGTT was performed after a 12-h overnight fast and plasma insulin and glucose concentrations were determined at 0, 30, 60, 90 and 120 min.

**Supplementary references**

1. Marmot M, Brunner E. Cohort Profile: the Whitehall II study. Int J Epidemiol 2005;34(2):251-6.

2. Alberti KG, Zimmet PZ. Definition, diagnosis and classification of diabetes mellitus and its complications. Part 1: diagnosis and classification of diabetes mellitus provisional report of a WHO consultation. Diabet Med 1998;15(7):539-53.

3. Miller GJ, Bauer KA, Barzegar S, Foley AJ, Mitchell JP, Cooper JA, et al. The effects of quality and timing of venepuncture on markers of blood coagulation in healthy middle-aged men. Thromb Haemost 1995;73(1):82-6.

4. Humphries SE, Gable D, Cooper JA, Ireland H, Stephens JW, Hurel SJ, et al. Common variants in the TCF7L2 gene and predisposition to type 2 diabetes in UK European Whites, Indian Asians and Afro-Caribbean men and women. J Mol Med 2006;84(12):1005-14.

5. Salpea KD, Gable DR, Cooper JA, Stephens JW, Hurel SJ, Ireland HA, et al. The effect of WNT5B IVS3C>G on the susceptibility to type 2 diabetes in UK Caucasian subjects. Nutr Metab Cardiovasc Dis 2009;19(2):140-5.

6. Elkeles RS, Feher MD, Flather MD, Godsland IF, Nugara F, Richmond W, et al. The association of coronary calcium score and conventional cardiovascular risk factors in Type 2 diabetic subjects asymptomatic for coronary heart disease (The PREDICT Study). Diabet Med 2004;21(10):1129-34.

7. Tiret L, Gerdes C, Murphy MJ, Dallongeville J, Nicaud V, O'Reilly DS, et al. Postprandial response to a fat tolerance test in young adults with a paternal history of premature coronary heart disease - the EARS II study (European Atherosclerosis Research Study). Eur J Clin Invest 2000;30(7):578-85.

**Supplemental Table 1. Details of the study cohorts used in the analysis.**

| **Study Name** | **Study Design (location)** | **Sampling frame** | **Main selection criteria** | **Number of individuals with DNA available** | **Traits available** | **SNPs genotyped**  **(number of individuals with SNP data)** |
| --- | --- | --- | --- | --- | --- | --- |
| Whitehall II  (WHII). | Population based prospective cohort (United Kingdom). | Men and women aged 35-55 yr, recruited from British Civil Servants from 20 London based offices. | All office based employees aged between 35-55 yr were invited to a cardiovascular screening at their workplace. The men and women recruited came from all 20 major government departments and were mainly administratively employed. | n=5,666 Caucasians; 5,235 with T2D data  n=226  Indian Asians; 200 with diabetic status, 148 used as diabetic-free controls for Indian Asian T2D patients in UDACS, EDS and PREDICT | Standard OGTT and HOMA-IR. Traits of interest in Supp Table 3. | rs2943641  (Caucasians: n=4,161;  302 with T2D at phase 7)  (Indian Asians: n=146)  rs6725556, rs16822570, rs16822573, rs16822574,  rs2435182, rs16822579, rs17208470, rs10182336, rs10181778, rs16822601,  rs16822604, rs7567312,  rs1078533, rs2435185,  rs16822626, rs16822630,  rs16822638, rs10498212,  rs4675094, rs10170579,  rs2288586, rs3769647,  rs3731594, rs1801278,  rs2229613, rs1801123, rs3731596, rs2234931,  rs1801118, rs6725330,  rs13423855, rs13018009,  rs4675096  (Caucasians: n=4,752;  345 with T2D at phase 7) |
| Northwick Park  Heart Study II (NPHSII). | Population-based prospective cohort  (United Kingdom). | All men aged between 50-64 yr, registered with 9 primary care practices. | Individuals excluded if had: pre-existing cardiovascular disease (CHD or stroke), coronary surgery or malignant disease, or were taking Aspirin or anticoagulant.  70 T2D patients at baseline excluded. | n=2,705 Caucasians | Traits of interest in Supp Table 3. | rs2943641  (n=2,642)  rs6725556  (n=2,625)  Either SNP:  (n=2,682)  (158 incident T2D cases) |
| University College London Diabetes and Cardiovascular Study (UDACS). | Disease cohort. Recruitment from the diabetic clinic at UCL hospital London between 2001-2002. | Men and women with T2D. | Diagnosis of T2D according to WHO criteria. | n=600 Caucasians  (59% men)  n=107  Indian Asians  (75% men) | Traits of interest in Supp Table 5. | rs2943641  (Caucasians: n=550;  Indian Asians: n=99)    rs6725556  (Caucasians: n=558;  Indian Asians: n=98)  Either SNP:  (Caucasians: n=570;  Indian Asians: n=101) |
| Ealing Diabetes Study (EDS). | Disease cohort. Recruitment from the diabetic clinic at Ealing hospital London between 2001-2002. | Men and women with T2D. | Diagnosis of T2D according to WHO criteria. | n=331 Caucasians  (60% men)  n=503  Indian Asians  (56% men) | Traits of interest in Supp Table 5. | rs2943641  (Caucasians: n=318;  Indian Asians: n=480)  rs6725556  (Caucasians: n=321;  Indian Asians: n=476)  Either SNP:  (Caucasians: n=326;  Indian Asians: n=488) |
| PRospective Evaluation of Diabetic Ischemic heart disease by Computer Tomography study (PREDICT). | Disease cohort. Recruitment from the diabetic clinic at several London hospitals between 2001-2002. | Men and women with T2D. | Diagnosis of T2D according to WHO criteria. Coronary artery calcification measured by computer tomography. Exclusion criteria: current or past history of CHD, congestive heart failure, uncontrolled hypertension, serious medical disorders likely to limit life expectancy or requiring extensive medical treatment. | n=400 Caucasians  (61% men)  n=118  Indian Asians  (74% men) | Traits of interest in Supp Table 5. | rs2943641  (Caucasians: n=375;  Indian Asians: n=112)  rs6725556  (Caucasians: n=354;  Indian Asians: n=101)  Either SNP:  (Caucasians: n=395;  Indian Asians: n=114) |
| European Arthrosclerosis Research Study (EARSII). | Offspring case control study  (European). | All men aged between 18-28 yr, recruited from 14 universities from 11 European countries. | ‘Cases’ recruited on basis of paternal early myocardial infarction (<55 yr).  Controls matched for age and recruitment centre. | n=390  ‘cases’  n=389  ‘controls’ | Standard OGTT,  HOMA-IR, other traits of interest in Supp Table 3. | rs2943641  (n=714)  rs6725556  (n=719)  Either SNP: (n=742) |

**Supplemental Table 2.** *IRS1*gene SNPs on the 50K HumanCVD BeadChip (Illumina) and their association with T2D in Whitehall-II.

| **SNP** | **Position**  **(NCBI36)** | **SNP type** | **Major allele** | **Minor allele** | **MAF**  **cases** | **MAF**  **controls** | **OR (95% CI)^a^** | ***p*-value^a^** | ***p*-value^b^** |
| --- | --- | --- | --- | --- | --- | --- | --- | --- | --- |
| rs16822570 | 227307777 | Exon2 (3’-UTR) | A | C | 0.030 | 0.052 | 0.57 (0.36-0.90) | **0.02** | **0.02** |
| rs16822573 | 227309497 | Intronic | C | A | 0.030 | 0.052 | 0.57 (0.36-0.90) | **0.02** | **0.02** |
| rs16822574 | 227310525 | Intronic | G | A | 0.030 | 0.052 | 0.57 (0.36-0.91) | **0.02** | **0.02** |
| rs2435182 | 227310874 | Intronic | A | G | 0 | 0 | - | - | - |
| rs16822579 | 227311744 | Intronic | G | C | 0 | 0 | - | - | - |
| rs17208470 | 227312149 | Intronic | C | A | 0.097 | 0.091 | 1.08 (0.83-1.40) | 0.59 | 0.39 |
| rs10182336 | 227317055 | Intronic | A | G | 0.046 | 0.074 | 0.61 (0.42-0.88) | **0.009** | **0.01** |
| rs10181778 | 227322481 | Intronic | T | A | 0.031 | 0.051 | 0.62 (0.39-0.97) | **0.04** | **0.04** |
| rs16822601 | 227324992 | Intronic | A | C | 0.013 | 0.026 | 0.50 (0.26-0.99) | **0.05** | 0.07 |
| rs16822604 | 227325979 | Intronic | G | A | 0.013 | 0.026 | 0.50 (0.26-0.98) | **0.05** | 0.07 |
| rs7567312 | 227327813 | Intronic | A | C | 0 | 0.0001 | - | - | - |
| rs1078533 | 227338831 | Intronic | C | A | 0.013 | 0.026 | 0.50 (0.26-0.98) | **0.05** | 0.07 |
| rs2435185 | 227344667 | Intronic | A | G | 0.024 | 0.027 | 0.89 (0.53-1.50) | 0.66 | 0.67 |
| rs16822626 | 227347128 | Intronic | C | G | 0 | 0 | - | - | - |
| rs16822630 | 227354699 | Intronic | G | A | 0.016 | 0.028 | 0.57 (0.31-1.05) | 0.07 | 0.11 |
| rs16822638 | 227356027 | Intronic | A | G | 0.056 | 0.073 | 0.77 (0.55-1.08) | 0.13 | 0.14 |
| rs10498212 | 227359519 | Intronic | A | T | 0.056 | 0.072 | 0.78 (0.56-1.09) | 0.15 | 0.14 |
| rs4675094 | 227360035 | Intronic | G | C | 0.068 | 0.093 | 0.72 (0.53-0.99) | **0.04** | **0.05** |
| rs10170579 | 227361479 | Intronic | C | G | 0 | 0 | - | - | - |
| rs2288586 | 227363634 | Intronic | G | C | 0.058 | 0.073 | 0.79 (0.56-1.10) | 0.16 | 0.16 |
| rs3769647 | 227367827 | Intronic | G | C | 0.046 | 0.063 | 0.73 (0.50-1.06) | 0.10 | 0.10 |
| rs3731594 | 227368290 | Exon1, ns-coding | G | A | 0 | 0 | - | - | - |
| rs1801278 | 227368788 | Exon1, ns-coding | G | A | 0.073 | 0.063 | 1.20 (0.88-1.63) | 0.25 | 0.35 |
| rs2229613 | 227368933 | Exon1, s-coding | G | A | 0 | 0.0003 | - | - | - |
| rs1801123 | 227369287 | Exon1, s-coding | A | G | 0.068 | 0.093 | 0.71 (0.52-0.97) | **0.03** | **0.04** |
| rs3731596 | 227370433 | Exon1, s-coding | A | G | 0 | 0 | - | - | - |
| rs2234931 | 227370997 | Exon1, s-coding | G | A | 0.074 | 0.063 | 1.21 (0.89-1.64) | 0.22 | 0.33 |
| rs1801118 | 227371073 | Exon1, ns-coding | A | G | 0 | 0 | - | - | - |
| rs6725330 | 227375101 | Upstream | A | G | 0.097 | 0.111 | 0.88 (0.67-1.14) | 0.33 | 0.40 |
| rs6725556 | 227375236 | Upstream | A | G | 0.044 | 0.070 | 0.62 (0.42-0.90) | **0.01** | **0.01** |
| rs13423855 | 227376537 | Upstream | G | A | 0 | 0.0001 | - | - | - |
| rs13018009 | 227376645 | Upstream | A | G | 0.016 | 0.023 | 0.73 (0.39-1.34) | 0.31 | 0.34 |
| rs4675096 | 227377185 | Upstream | G | A | 0.089 | 0.084 | 1.07 (0.81-1.41) | 0.63 | 0.65 |

Odd ratios (OR) and 95% confidence intervals (CI) are for an additive effect (effect per minor allele). ^a^Adjusted for age and gender; ^b^additionally adjusted for body mass index. ns-coding = non synonymous coding; s-coding = synonymous coding; MAF = minor allele frequency.

**Supplemental Table 3.** Baseline clinical, biochemical and genetic characteristics of study participants in WHII, NPHSII and EARSII studies.

|  | ***WHII*** | |  | ***NPHSII*** | |  | ***EARSII*** | |  |
| --- | --- | --- | --- | --- | --- | --- | --- | --- | --- |
|  | Non diabetic | With T2D | *p-*value | Non diabetic | With T2D | *p-*value | Control | Cases | *p-*value |
| Number (n) | 4,407 | 345 |  | 2,524 | 158 |  | 369 | 345 |  |
| Males, % (n) | 73.8 (3,252) | 73.3 (253) | 0.85 | 100.0 (2,524) | 100.0 (158) | NA | 100.0 (369) | 100.0 (345) | NA |
| Age (yr) | 43.6 (5.9) | 45.8 (6.2) | <0.001 | 56.0 (3.4) | 56.3 (3.4) | 0.38 | 22.8 (2.7) | 22.9 (2.8) | 0.70 |
| Body mass index (kg/m^2^) | 24.2 (3.1) | 26.6 (4.1) | <0.001 | 26.1 (3.3) | 28.5 (3.6) | <0.001 | 23.1 (2.7) | 23.2 (2.9) | 0.48 |
| Obesity, % (n) | 4.1 (181) | 19.5 (67) | <0.001 | 12.3 (311) | 33.1 (52) | <0.001 | 2.5 (9) | 3.2 (11) | 0.54 |
| Systolic BP (mmHg) | 121.9 (13.7) | 128.3 (14.9) | <0.001 | 136.7 (18.6) | 142.1 (19.2) | 0.001 | 117.0 (10.8) | 117.3 (10.9) | 0.76 |
| Diastolic BP (mmHg) | 75.3 (9.7) | 80.3 (10.2) | <0.001 | 84.5 (11.2) | 86.4 (11.3) | 0.04 | 73.3 (9.9) | 73.7 (11.1) | 0.60 |
| Hypertension, % (n) | 16.4 (723) | 33.4 (115) | <0.001 | 51.7 (1,303) | 63.9 (101) | 0.003 | 7.9 (29) | 9.3 (32) | 0.46 |
| Current smokers, % (n) | 14.2 (620) | 17.8 (61) | 0.07 | 28.1 (710) | 34.8 (55) | 0.07 | 7.6 (28) | 8.7 (30) | 0.59 |
| Cholesterol (mmol/l) | 5.76 (1.09) | 6.10 (1.07) | <0.001 | 5.73 (1.01) | 5.90 (0.98) | 0.04 | 4.22 (0.78) | 4.45 (0.87) | <0.001 |
| Glucose (mmol/l) | 5.18 (0.45) | 5.89 (1.46) | <0.001 | - | - | - | 5.18 (0.41) | 5.19 (0.44) | 0.68 |
| HbA1c (%) | 5.19 (0.37) | 6.49 (1.20) | <0.001 | - | - | - | - | - | - |
| Insulin (pmol/l) | 34.2 (22.4) | 62.9 (48.6) | <0.001 |  |  |  | 81.3 (33.3) | 75.7 (33.3) | 0.04 |
| HOMA-IR | 1.13 (0.78) | 2.30 (1.96) | <0.001 |  |  |  | 2.83 (1.12) | 2.64 (1.05) | 0.03 |
| rs2943641genotype, % (n)* |  |  |  |  |  |  |  |  |  |
| CC | 41.9 (1,616) | 47.4 (143) |  | 42.3 (1,052) | 40.5 (62) |  | 42.8 (158) | 44.4 (153) |  |
| CT | 45.2 (1,745) | 41.1 (124) | 0.18 | 46.1 (1,148) | 48.4 (74) | 0.86 | 44.4 (164) | 45.2 (156) | 0.63 |
| TT | 12.9 (498) | 11.6 (35) |  | 11.6 (289) | 11.1 (17) |  | 12.7 (47) | 10.4 (36) |  |
| T allele (95% CI) | 0.36  (0.34-0.37) | 0.32  (0.28-0.36) | 0.09 | 0.35  (0.33-0.36) | 0.35  (0.30-0.41) | 0.83 | 0.35  (0.32-0.39) | 0.33  (0.30-0.37) | 0.45 |

Data are presented as mean values (±SD) or as percentages (numbers in parenthesis). All blood measures were adjusted for age, body mass index (BMI) and smoking, plus gender in WHII study. Obesity was defined as BMI ≥30 kg/m^2^; Hypertension was defined as systolic/diastolic BP values ≥140/90 mmHg or use of anti-hypertensive treatment. Cases in EARSII are subjects whose father had a proven myocardial infarction before the age of 55 yr.

*Available for 3,859 non-diabetic and 302 T2D subjects in WHII and for 2,489 non-diabetic and 153 T2D subjects in NPHSII.

Abbreviations: T2D = type-2 diabetes; BP = blood pressure; HbA1c = glycated haemoglobin; HOMA-IR = homeostasis model assessment index of insulin resistance; CI = confidence interval; WHII = Whitehall-II; NPHSII = Northwick Park Heart Study-II; EARSII = European Atherosclerosis Research Study-II.

**Supplemental Table 4.** Odds ratios (95% CI) for T2D and mixed regression models for glucose, insulin and HOMA-IR over all phases in Whitehall-II for SNP rs2943641 and for 23 *IRS1* SNPs on HumanCVD BeadChip. ORs and B (SE) are for an additive effect (effect per minor allele) adjusted for age, gender, body mass index and study phase.

|  | **rs2943641** | | **rs16822570** | | **rs16822573** | | **rs16822574** | |
| --- | --- | --- | --- | --- | --- | --- | --- | --- |
| *Trait* | *B (SE)* | *p-value* | *B (SE)* | *p-value* | *B (SE)* | *p-value* | *B (SE)* | *p-value* |
| Fasting glucose^a^ | -0.004 (0.002) | 0.13 | -0.005 (0.005) | 0.31 | -0.005 (0.005) | 0.32 | -0.005 (0.005) | 0.31 |
| 2 hour glucose^a^ | -0.003 (0.005) | 0.54 | -0.003 (0.011) | 0.81 | -0.003 (0.011) | 0.81 | -0.003 (0.011) | 0.76 |
| Fasting insulin^a^ | -0.022 (0.011) | **0.04** | -0.014 (0.022) | 0.52 | -0.014 (0.022) | 0.52 | -0.013 (0.022) | 0.55 |
| 2 hour insulin^a^ | -0.010 (0.015) | 0.48 | 0.028 (0.031) | 0.36 | 0.029 (0.031) | 0.35 | 0.028 (0.031) | 0.37 |
| HOMA-IR^a^ | -0.026 (0.012) | **0.03** | -0.020 (0.024) | 0.42 | -0.020 (0.024) | 0.42 | -0.019 (0.024) | 0.44 |
| OR (95% CI) | 0.82 (0.69-0.99) | **0.04** | 0.58 (0.36-0.92) | **0.02** | 0.58 (0.36-0.92) | **0.02** | 0.58 (0.36-0.92) | **0.02** |

|  | **rs17208470** | | **rs10182336** | | **rs10181778** | | **rs16822601** | |
| --- | --- | --- | --- | --- | --- | --- | --- | --- |
| *Trait* | *B (SE)* | *p-value* | *B (SE)* | *p-value* | *B (SE)* | *p-value* | *B (SE)* | *p-value* |
| Fasting glucose^a^ | 0.003 (0.004) | 0.44 | -0.004 (0.004) | 0.34 | -0.004 (0.005) | 0.40 | -0.011 (0.007) | 0.13 |
| 2 hour glucose^a^ | -0.009 (0.008) | 0.29 | -0.010 (0.009) | 0.26 | -0.004 (0.011) | 0.68 | -0.020 (0.015) | 0.19 |
| Fasting insulin^a^ | -0.015 (0.017) | 0.38 | 0.003 (0.019) | 0.88 | -0.009 (0.022) | 0.69 | -0.002 (0.032) | 0.69 |
| 2 hour insulin^a^ | -0.030 (0.023) | 0.19 | 0.018 (0.026) | 0.49 | 0.020 (0.031) | 0.51 | -0.002 (0.044) | 0.97 |
| HOMA-IR^a^ | -0.011 (0.018) | 0.54 | -0.000 (0.0251) | 0.99 | -0.013 (0.024) | 0.60 | -0.007 (0.034) | 0.83 |
| OR (95% CI) | 1.12 (0.86-1.47) | 0.39 | 0.62 (0.42-0.90) | **0.01** | 0.62 (0.39-0.98) | **0.04** | 0.53 (0.27-1.04) | 0.07 |

|  | **rs16822604** | | **rs1078533** | | **rs2435185** | | **rs16822630** | |
| --- | --- | --- | --- | --- | --- | --- | --- | --- |
| *Trait* | *B (SE)* | *p-value* | *B (SE)* | *p-value* | *B (SE)* | *p-value* | *B (SE)* | *p-value* |
| Fasting glucose^a^ | -0.010 (0.007) | 0.17 | -0.010 (0.007) | 0.17 | -0.001 (0.007) | 0.89 | -0.009 (0.007) | 0.19 |
| 2 hour glucose^a^ | -0.017 (0.015) | 0.27 | -0.017 (0.015) | 0.27 | 0.032 (0.015) | **0.03** | -0.016 (0.015) | 0.26 |
| Fasting insulin^a^ | -0.001 (0.031) | 0.97 | -0.001 (0.031) | 0.97 | 0.017 (0.030) | 0.57 | 0.011 (0.030) | 0.71 |
| 2 hour insulin^a^ | 0.011 (0.044) | 0.80 | 0.011 (0.044) | 0.80 | 0.110 (0.042) | **0.01** | 0.022 (0.041) | 0.59 |
| HOMA-IR^a^ | -0.006 (0.034) | 0.86 | -0.006 (0.034) | 0.86 | 0.021 (0.033) | 0.52 | 0.007 (0.033) | 0.83 |
| OR (95% CI) | 0.53 (0.27-1.04) | 0.07 | 0.53 (0.27-1.04) | 0.07 | 0.89 (0.52-1.51) | 0.67 | 0.60 (0.32-1.11) | 0.11 |

**Supplemental Table 4** continued

|  | **rs16822638** | | **rs10498212** | | **rs4675094** | | **rs2288586** | |
| --- | --- | --- | --- | --- | --- | --- | --- | --- |
| *Trait* | *B (SE)* | *p-value* | *B (SE)* | *p-value* | *B (SE)* | *p-value* | *B (SE)* | *p-value* |
| Fasting glucose^a^ | -0.007 (0.004) | 0.12 | -0.007 (0.004) | 0.10 | -0.006 (0.004) | 0.14 | -0.007 (0.004) | 0.11 |
| 2 hour glucose^a^ | 0.003 (0.009) | 0.78 | 0.001 (0.009) | 0.89 | -0.002 (0.008) | 0.81 | 0.002 (0.009) | 0.82 |
| Fasting insulin^a^ | 0.002 (0.019) | 0.90 | -0.005 (0.019) | 0.81 | 0.003 (0.017) | 0.88 | 0.001 (0.019) | 0.96 |
| 2 hour insulin^a^ | 0.037 (0.026) | 0.15 | 0.030 (0.026) | 0.25 | 0.034 (0.023) | 0.14 | 0.036 (0.026) | 0.16 |
| HOMA-IR^a^ | -0.003 (0.020) | 0.87 | -0.011 (0.020) | 0.60 | -0.001 (0.018) | 0.94 | -0.005 (0.020) | 0.81 |
| OR (95% CI) | 0.77 (0.55-1.09) | 0.14 | 0.77 (0.55-1.09) | 0.14 | 0.73 (0.54-1.01) | **0.05** | 0.78 (0.56-1.10) | 0.16 |

|  | **rs3769647** | | **rs1801278** | | **rs1801123** | | **rs2234931** | |
| --- | --- | --- | --- | --- | --- | --- | --- | --- |
| *Trait* | *B (SE)* | *p-value* | *B (SE)* | *p-value* | *B (SE)* | *p-value* | *B (SE)* | *p-value* |
| Fasting glucose^a^ | -0.005 (0.005) | 0.27 | 0.005 (0.005) | 0.32 | -0.006 (0.004) | 0.14 | 0.005 (0.004) | 0.24 |
| 2 hour glucose^a^ | 0.001 (0.010) | 0.96 | -0.010 (0.010) | 0.31 | -0.003 (0.008) | 0.70 | -0.010 (0.010) | 0.33 |
| Fasting insulin^a^ | -0.001 (0.020) | 0.95 | -0.003 (0.020) | 0.90 | 0.005 (0.017) | 0.78 | 0.004 (0.020) | 0.85 |
| 2 hour insulin^a^ | 0.035 (0.028) | 0.20 | -0.046 (0.028) | 0.09 | 0.035 (0.023) | 0.14 | -0.044 (0.028) | 0.11 |
| HOMA-IR^a^ | -0.005 (0.022) | 0.81 | 0.002 (0.022) | 0.93 | 0.000 (0.018) | 0.98 | 0.009 (0.022) | 0.69 |
| OR (95% CI) | 0.73 (0.50-1.06) | 0.10 | 1.16 (0.85-1.60) | 0.35 | 0.72 (0.53-0.99) | **0.04** | 1.17 (0.85-1.60) | 0.33 |

|  | **rs6725330** | | **rs6725556** | | **rs13018009** | | **rs4675096** | |
| --- | --- | --- | --- | --- | --- | --- | --- | --- |
| *Trait* | *B (SE)* | *p-value* | *B (SE)* | *p-value* | *B (SE)* | *p-value* | *B (SE)* | *p-value* |
| Fasting glucose^a^ | -0.002 (0.004) | 0.59 | -0.007 (0.004) | 0.10 | -0.003 (0.007) | 0.64 | -0.002 (0.004) | 0.66 |
| 2 hour glucose^a^ | -0.011 (0.008) | 0.16 | -0.005 (0.010) | 0.57 | 0.005 (0.016) | 0.75 | -0.009 (0.009) | 0.30 |
| Fasting insulin^a^ | -0.004 (0.016) | 0.80 | 0.015 (0.019) | 0.43 | 0.067 (0.033) | 0.04 | 0.012 (0.017) | 0.49 |
| 2 hour insulin^a^ | -0.008 (0.022) | 0.72 | 0.045 (0.027) | 0.09 | 0.029 (0.047) | 0.53 | -0.027 (0.024) | 0.27 |
| HOMA-IR^a^ | -0.005 (0.017) | 0.75 | 0.010 (0.021) | 0.65 | 0.067 (0.036) | 0.06 | 0.010 (0.019) | 0.60 |
| OR (95% CI) | 0.89 (0.68-1.17) | 0.40 | 0.60 (0.41-0.89) | **0.01** | 0.74 (0.40-1.38) | 0.34 | 1.07 (0.80-1.42) | 0.65 |

^a^ B (SE) is on log scale

**Supplemental Table 5.** Baseline clinical, biochemical and genetic characteristics of T2D cases by ethnic group in UDACS, EDS and PREDICT studies combined.

|  | ***European Whites***  ***with T2D*** | ***Indian Asians***  ***with T2D*** |
| --- | --- | --- |
| Number (n) | 1,291 | 703 |
| Males, % (n) | 60.5 (778) | 61.5 (430) |
| Age (yr) | 64.9 (10.9) | 58.7 (10.9) |
| Age of onset (yr) | 54.9 (12.1) | 47.9 (11.2) |
| Duration of diabetes (yr) | 8.17 (7.6) | 9.08 (7.6) |
| Body mass index (kg/m^2^) | 29.4 (5.4) | 27.4 (4.4) |
| Obesity, % (n) | 45.6 (574) | 26.8 (176) |
| Systolic BP (mmHg) | 136.8 (19.8) | 134.1 (20.5) |
| Diastolic BP (mmHg) | 77.6 (11.1) | 76.5 (11.2) |
| Hypertension, % (n) | 74.9 (924) | 67.3 (459) |
| Current smokers, % (n) | 16.7 (213) | 7.9 (55) |
| Cholesterol (mmol/l) | 4.86 (1.05) | 4.76 (1.10) |
| Glucose (mmol/l) | 10.3 (4.2) | 10.8 (4.4) |
| HbA1c (%) | 8.01 (1.76) | 8.76 (2.02) |
| rs2943641 genotype , % (n)* |  |  |
| CC | 45.2 (562) | 59.6 (412) |
| CT | 42.8 (532) | 34.5 (238) |
| TT | 12.0 (149) | 5.9 (41) |
| T allele (95% CI) | 0.33 (0.32-0.35) | 0.23 (0.21-0.25) |

Data are presented as mean values (±SD) or as percentages (numbers in parenthesis). Obesity was defined as BMI ≥30 kg/m^2^; Hypertension was defined as systolic/diastolic BP values ≥140/90 mmHg or use of anti-hypertensive treatment.

*Available for 1,243 European whites and 691 Indian Asians.

Abbreviations: T2D = type-2 diabetes; BP = blood pressure; HbA1c = glycated haemoglobin; CI = confidence interval; UDACS = University College London Diabetes and Cardiovascular Study; EDS = Ealing Diabetes Study; PREDICT = PRospective Evaluation of Diabetic Ischemic heart disease by Computed Tomography.

**Supplemental Table 6.** Characteristics of the study T2D cases according to rs2943641 genotype in European Whites and Indian Asians of the UDACS, EDS and PREDICT studies combined.

|  | ***European Whites with T2D*** | | | | ***Indian Asians with T2D*** | | | |
| --- | --- | --- | --- | --- | --- | --- | --- | --- |
|  | CC | CT | TT | *p-*value | CC | CT | TT | *p-*value |
| Number (n) | 562 | 532 | 149 |  | 412 | 238 | 41 |  |
| Age of onset (yr) | 55.2 (12.1) | 54.6 (12.1) | 54.5 (12.2) | 0.68 | 47.8 (11.3) | 48.7 (11.4) | 45.8 (11.3) | 0.25 |
| Body mass index (kg/m^2^) | 29.1 (5.4) | 29.4 (5.5) | 30.8 (5.7) | 0.002 | 27.2 (4.5) | 27.6 (4.5) | 28.4 (4.6) | 0.17 |
| Cholesterol (mmol/l) | 4.97 (1.08) | 4.97  (1.07) | 4.94 (1.08) | 0.95 | 4.88 (1.12) | 4.95 (1.13) | 4.74 (1.13) | 0.47 |
| Glucose (mmol/l) | 10.06 (4.01) | 10.46 (4.17) | 10.39 (4.17) | 0.24 | 10.81 (4.22) | 11.03 (4.32) | 10.48 (4.29) | 0.68 |
| HbA1c (%) | 7.99 (1.75) | 8.00 (1.75) | 8.08 (1.76) | 0.85 | 8.82 (4.09) | 8.66 (4.10) | 8.84 (4.08) | 0.89 |

Data are presented as mean (±SD).

Abbreviations: T2D = type-2 diabetes; HbA1c = glycated haemoglobin; UDACS = University College London Diabetes and Cardiovascular Study; EDS = Ealing Diabetes Study; PREDICT = Prospective Evaluation of Diabetic Ischemic heart disease by Computed Tomography.

**Supplemental Table 7.** Glucose and insulin levels after an oral glucose tolerance test and HOMA-IR by rs2943641 genotype in EARSII (‘cases’ and ‘controls’ combined).

**Glucose (mmol/l)**

| rs2943641 genotype | 0 min | 30 min | 60 min | 90 min | 120 min | AUC |
| --- | --- | --- | --- | --- | --- | --- |
| CC | 5.21 (0.02) | 7.74 (0.08) | 6.32 (0.10) | 5.46 (0.07) | 4.87 (0.06) | 1.85 (0.11) |
| CT | 5.17 (0.02) | 7.66 (0.08) | 6.05 (0.10) | 5.30 (0.07) | 4.75 (0.06) | 1.64 (0.11) |
| TT | 5.12 (0.05) | 7.63 (0.15) | 6.09 (0.19) | 5.41 (0.14) | 4.83 (0.12) | 1.81 (0.21) |
| *p-*value^a^ | 0.07 | 0.44 | 0.10 | 0.34 | 0.40 | 0.43 |

**Insulin (pmol/l)^e^**

| rs2943641 genotype | 0 min | 30 min | 60 min | 90 min | 120 min | AUC |
| --- | --- | --- | --- | --- | --- | --- |
| CC | 79.1 (1.9) | 399.7 (11.3) | 339.9 (11.0) | 249.4 (8.0) | 161.4 (6.7) | 401.7 (12.4) |
| CT | 77.2 (1.9) | 380.0 (11.0) | 298.1 (10.3) | 219.6 (7.5) | 146.1 (6.4) | 356.3 (11.6) |
| TT | 82.0 (3.9) | 382.5 (21.6) | 290.3 (19.9) | 226.6 (15.0) | 143.6 (12.4) | 346.0 (22.4) |
| *p-*value^a^ | 0.86 | 0.28 | 0.004 | 0.03 | 0.09 | 0.005 |
| *p-*value^b^ | NA | 0.24 | 0.002 | 0.02 | 0.07 | 0.004 |
| *p-*value^c^ | 0.49^d^ | 0.33 | 0.004 | 0.02 | 0.07 | 0.008 |

HOMA-IR

| rs2943641 genotype | 0 min | *p-*value^a^ |  |  |  |  |
| --- | --- | --- | --- | --- | --- | --- |
| CC | 2.63 (0.07) |  |  |  |  |  |
| CT | 2.56 (0.07) | 0.95 |  |  |  |  |
| TT | 2.68 (0.13) |  |  |  |  |  |

Results are mean ± SEM.

^a^ *p*-values adjusted for age, case/control status and region.

^b^ *p*-values adjusted for age, case/control status, region and fasting insulin level.

^c^ *p*-values adjusted for age, case/control status, region, fasting insulin level and body mass index.

^d^ adjusted for age, case/control status, region and body mass index.

^e^ Transformed back from square root transformed data.

Abbreviations: EARSII = European Atherosclerosis Research Study-II; AUC = area under the curve; HOMA-IR = homeostasis model assessment index of insulin resistance.

**Supplemental Table 8.** Genotype distribution for *IRS1* SNP rs672556 and OR (95% CI) for T2D in all study cohorts. ORs are for an additive effect (effect per minor G-allele).

| **Study cohort** | **rs6725556 genotype** | **Non-diabetic subjects** | **With T2D** | **OR (95% CI)^a^** | ***p*-value^a^** | ***p*-value^b^** |
| --- | --- | --- | --- | --- | --- | --- |
|  |  |  |  |  |  |  |
| WHII | AA | 3,716 (86.4) | 309 (91.4) | 0.62 (0.42-0.90) | 0.01 | 0.01 |
|  | AG | 569 (13.2) | 28 (8.3) |  |  |  |
|  | GG | 18 (0.4) | 1 (0.3) |  |  |  |
|  | G allele (95% CI) | 0.070 (0.065-0.076) | 0.044 (0.030-0.063) |  |  |  |
|  |  |  |  |  |  |  |
| NPHSII | AA | 2,144 (86.8) | 137 (89.0) | 0.80 (0.50-1.30) | 0.37 | 0.65 |
|  | AG | 308 (12.5) | 17 (11.0) |  |  |  |
|  | GG | 19 (0.8) | 0 (0) |  |  |  |
|  | G allele (95% CI) | 0.070 (0.063-0.077) | 0.055 (0.032-0.087) |  |  |  |
|  |  |  |  |  |  |  |
| European White T2D cases^c^ | AA | 2,144 (86.8) | 1,079 (87.5) | 0.89 (0.71-1.12) | 0.33 | 0.85 |
|  | AG | 308 (12.5) | 149 (12.1) |  |  |  |
|  | GG | 19 (0.8) | 5 (0.4) |  |  |  |
|  | G allele (95% CI) | 0.070 (0.063-0.077) | 0.064 (0.055-0.075) |  |  |  |
|  |  |  |  |  |  |  |
| Indian Asian  T2D cases^c^ | AA | 107 (77.0) | 529 (78.3) | 0.86 (0.59-1.26) | 0.44 | - |
|  | AG | 27 (19.4) | 135 (20.1) |  |  |  |
|  | GG | 5 (3.6) | 11 (1.6) |  |  |  |
|  | G allele (95% CI) | 0.138 (0.099-0.185) | 0.117 (0.010-0.135) |  |  |  |

^a^Adjusted for age and were applicable for gender and recruitment centre; ^b^additionally adjusted for body mass index.

^c^T2D cases of the UDACS, EDS and PREDICT studies combined (see Supplementary Table 1).

Abbreviations: T2D = type-2 diabetes; SNP = single nucleotide polymorphism; OR = Odds ratio; CI = confidence interval; WHII = Whitehall-II; NPHSII = Northwick Park Heart Study-II.

**Supplemental Table 9.**

1. Genotype distribution for *IRS1* SNP rs1801278 (Gly972Arg) and OR (95% CI) for T2D stratified by age in Whitehall-II study.

|  | **Age <=60 years** | | **Age >60 years** | |
| --- | --- | --- | --- | --- |
| **Rs1801278** | **Non-diabetic** | **With T2D** | **Non-diabetic** | **With T2D** |
| 11  12  22 | 1,987 (87.8)  267 (11.8)  8 (0.4) | 96 (82.1)  20 (17.1)  1 (0.9) | 1,779 (87.7)  246 (12.1)  4 (0.2) | 190 (87.6)  27 (12.4)  0 (0) |
| OR (95% CI) | 1.00 | 1.56 (0.99-2.47) | 1.00 | 0.99 (0.65-1.51) |

Association is greater in younger participants (OR=1.56 in younger participants vs. OR=0.99 in older participants; *p*-interaction =0.15).

1. Odds ratios by age of onset for T2D for rs2943641 and rs6725556 in the European Whites.

| **Rs2943641** | **Non-diabetic** | **Onset before 56 years** | **Onset after 55 years** |
| --- | --- | --- | --- |
| 11  12  22 | 1,052 (42.3)  1,148 (46.1)  289 (11.6) | 269 (43.1)  275 (44.1)  80 (12.8) | 291 (47.3)  257 (41.8)  67 (10.9) |
| OR (95% CI) | 1.00 | 1.01 (0.88-1.15) | 0.88 (0.77-1.00) |

| **Rs6725556** | **Non-diabetic** | **Onset before 56 years** | **Onset after 55 years** |
| --- | --- | --- | --- |
| 11  12  22 | 2,144 (86.7)  308 (12.5)  19 (0.8) | 543 (89.2)  65 (10.7)  1 (0.2) | 517 (86.0)  80 (13.3)  4 (0.7) |
| OR (95% CI) | 1.00 | 0.78 (0.60-1.02) | 1.05 (0.83-1.33) |

**Supplemental Figure 1.** Gene map and linkage disequilibrium (LD) plot (Haploview, version 4.1) of *IRS1* SNPs (given below the gene map) in Whitehall-II study. Values represent r^2^. LD is observed across the gene region with r^2^ values ranging between 0.0 and 1.0.


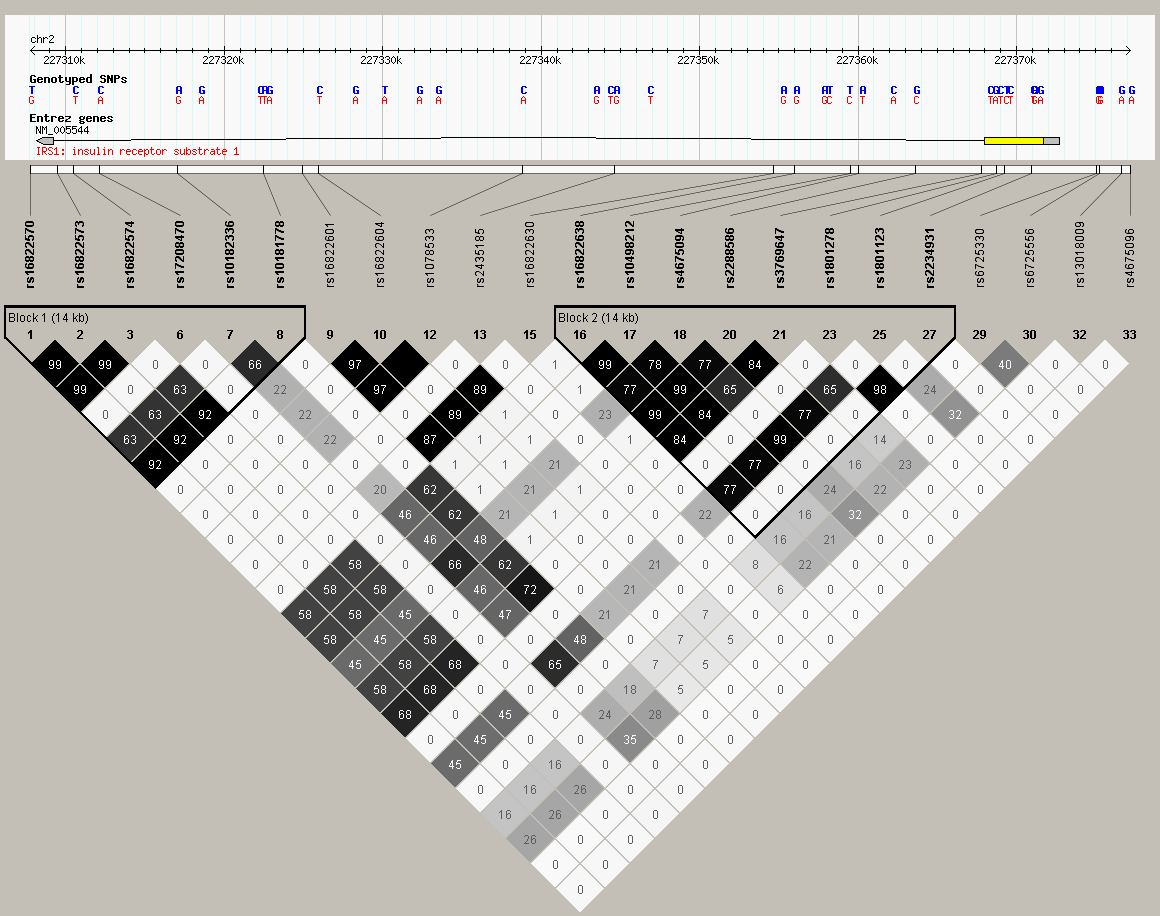


**Supplementary Information**

**The EARS II Group Collaborating Centres and their Associated Investigators:**

**Austria:** H. J. Menzel, Institute for Medical Biology and Genetics, University of Innsbruck, laboratory.

**Belgium:** G. De Backer, S. De Henauw, Department of Public Health, University of Ghent, recruitment centre.

**Belgium:** M. Rosseneu, Laboratorium voor Lipoproteïne Chemie/Vakgroep Biochemie, University of Ghent, laboratory.

**Denmark:** O. Faergeman, C. Gerdes, Medical Department I, Aarhus Amtssygehus, Aarhus, recruitment centre.

**Estonia:** M. Saava, K. Aasvee, Department of Nutrition and Metabolism, Estonian Institute of Cardiology, Tallinn, recruitment centre.

**Finland:** C. Ehnholm*, R. Elovainio**, J. Peräsalo, *National Public Health Institute, **The Finnish Student Health Service, Helsinki, recruitment centre.

**Finland**: Y.A. Kesäniemi*, M.J. Savolainen*, P. Palomaa**, *Department of Internal Medicine and Biocenter, Oulu, **The Finnish Student Health Service, University of Oulu, recruitment centre.

**France:** L. Tiret, V. Nicaud, O. Poirier, INSERM U525, Paris, EARS data centre, laboratory.

**France:** S. Visvikis, Centre de Médecine Préventive, Nancy, laboratory.

**France:** J.C. Fruchart, J. Dallongeville, Service de Recherche sur les Lipoprotéines et l'Athérosclérose (SERLIA), INSERM U325, Institut Pasteur, Lille, laboratory.

**Germany:** U. Beisiegel, C. Dingler, Medizinische Klinik Universitäts-Krankenhaus Eppendorf, Hamburg, recruitment centre and laboratory.

**Greece:** G. Tsitouris, N. Papageorgakis, G. Kolovou, Department of Cardiology, Evangelismos Hospital, Athens, recruitment centre.

**Italy:** E. Farinaro, Dept. of Medical Preventive Sciences, University "Frederico II" of Naples, recruitment centre.

**The Netherlands:** L.M. Havekes, IVVO-TNO Health Research, Gaubius Institute, Leiden, laboratory.

**Portugal:** M.J. Halpern, J. Canena, Instituto Superior de Ciencas da Saude, Lisbon, recruitment centre.

**Spain:** L. Masana, J. Ribalta, A. Jammoul, A. LaVille, Unitat Recerca Lipids, University Rovira i Virgili, Reus, recruitment centre and laboratory.

**Switzerland:** F. Gutzwiller, B. Martin, Institute of Social and Preventive Medicine, University of Zurich, recruitment centre and laboratory.

**United Kingdom:** D. St J. O'Reilly, M. Murphy, Institute of Biochemistry, Royal Infirmary, Glasgow, recruitment centre and laboratory.

**United Kingdom:** S.E. Humphries, P.J. Talmud, V. Gudnason, R.M. Fisher, University College London School of Medicine, London, laboratory.

**United Kingdom:** D. Stansbie, A.P. Day, M. Edgar, Department of Chemical Pathology, Royal Infirmary, Bristol, recruitment centre and laboratory.

**United Kingdom:** F. Kee*, A. Evans**, *Northern Health and Social Services Board, **Department of Epidemiology and Public Health, the Queen's University of Belfast, Belfast, recruitment centre.
